# Painful transient edema in the tibial diaphysis: the challenges and the essence of biopsy in treatment

**DOI:** 10.1186/s12957-018-1405-7

**Published:** 2018-06-08

**Authors:** Yavuz Arikan, Yasar Mahsut Dincel, Baris Ozkul, Rasit Ozcafer, Akay Kirat, Devrim Ozer

**Affiliations:** Orthopedic Surgeon, Department of Orthopedics and Traumatology, Metin Sabancı Baltalimani Bone and Joint Diseases Training and Research Hospital, Istanbul, Turkey

**Keywords:** Bone, Edema, Tibia, Transient, Treatment

## Abstract

**Background:**

Some patients experience a non-traumatic pain in the tibial diaphysis similar to that in the clinical and radiological findings of a tumor, an infection or a stress fracture and cannot be definitively diagnosed even after biopsy.

In this study, our aim was to exhibit the challenges in the diagnosis of this patient group and to evaluate this type of patients with a limited population in the literature.

**Methods:**

Eighteen extremities of 16 patients, whose complaints of non-traumatic pain in the tibial diaphysis were evaluated by our tumor council and T2-weighted MR scans of the medullary bone had shown hyperintense signal changes or tumor-like appearances, were evaluated with histological, radiological, and clinical results.

**Results:**

Lesions were detected in 18 extremities of the 16 patients (seven males, nine females; mean age 23 [range 7 to 51] years). Four of the lesions were in the right tibial diaphysis, ten were in the left, and two were bilateral. Laboratory findings of the patients were normal. Based on the decision of the tumor council, biopsy was performed on 12 patients. All patients’ complaints were gone and MRI findings decreased during the follow-up period. The complaints of the three patients who did not have a biopsy decreased after a mean period of three months.

**Conclusions:**

Medullary stress syndrome has been reported in the literature in various forms and in a limited number of cases, including longitudinal stress fracture and transient medullary edema of the bone. In light of our findings, we deduced that biopsy of the diaphyseal lesions in this patient group is essential and that the complaints of this patient group declined in the earlier term in comparison to the patients who were not performed biopsy.

## Background

Bone marrow edema is a condition that has been described following the routine use of magnetic resonance imaging (MRI). The condition may emerge due to several reasons, varying from traumatic damages on the bone to tumoral conditions. Therefore, the differential diagnosis of the condition is of essence. Transient edema of the bone is a pathology defined by different terms and was first described by Curtiss and Kincaid in 1959 as a clinical syndrome characterized by hip pain, loss of bone density in direct radiographs, and spontaneous healing in the last trimester of pregnancy [1].

Bone marrow edema may occur due to trauma-induced lesions, degenerative lesions, inflammatory lesions, ischemic lesions, infectious lesions, metabolic/endocrine lesions, iatrogenic lesions, or neoplastic causes [2].

Patients with bone tenderness are expected to have either normal appearance or non-specific radiographic changes in conventional radiographs, while those with an increased signal intensity in their MR scans can be considered to have an infection or neoplasm [3]. Moreover, stress fractures may also cause an increase in bone tenderness and signal intensity in the MR scans. The fracture line may not be detected in radiographs but is distinguished in computed tomography (CT) [4] and MR scans [5].

In our study, we aimed to evaluate the challenges in the diagnosis and treatment of the patient group with pain in the tibial diaphysis and bone marrow edema, with clinical and radiological findings similar to those of a tumor, osteomyelitis and stress fracture, and that are reported over a limited population in the literature.

## Methods

Patients with the presence of a non-traumatic tibial tenderness and pain, radiological or histological findings distinguishing the condition from a stress fracture, a tumor, an infection, absence of a fracture line in CT scans, presence of an uptake in bone scintigraphy, or a diaphyseal segmental bone marrow edema in MR scans met the inclusion criteria of our study.

Eighteen tibias of the 16 patients, who had presented to our clinic with complaints of severe non-traumatic tibial pain and had shown no symptoms in conventional radiographs but hyperintense signal changes or tumor-like appearances in the medullary bone in T2-weighted MR scans, were evaluated. No stress fracture or infection was detected in the tibias, and the laboratory results were normal.

**Fig. 1:**
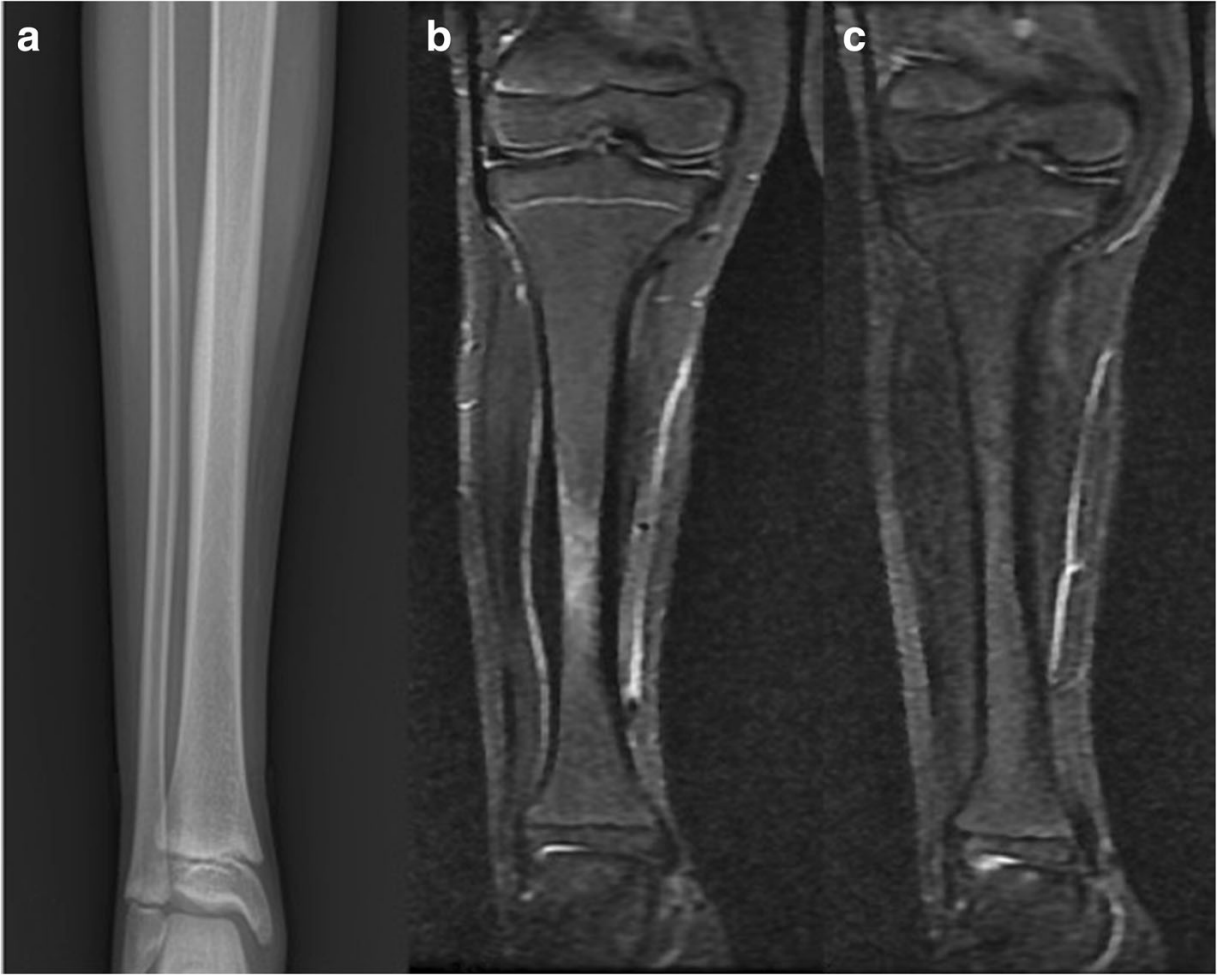
**a** Conventional radiograph of the 8-year-old patient diagnosed with transient edema in the tibia. **b** Preoperative coronal T2-weighted MR image. **c** Postoperative T2-weighted MR image

**Fig. 2:**
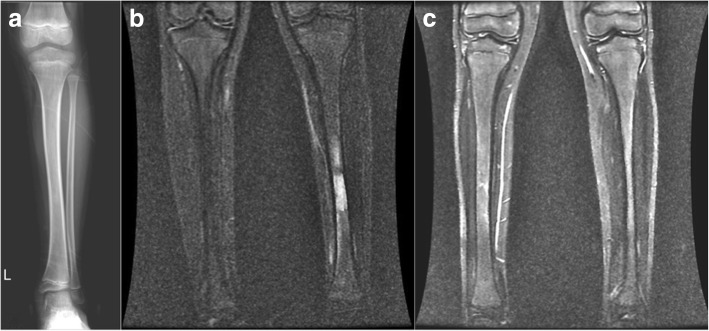
**a** Conventional radiograph of the 7-year-old patient diagnosed with transient edema in the tibia. **b** Coronal T2-weighted MR image. **c** T2-weighted MR image

The mean age of the 16 patients (seven males, nine females) was 23 (range 7 to 51) years. A total of 18 lesions were detected in the diaphyseal region; four lesions were located in the right tibia, ten were in the left, and two were bilateral (Table 1, Figs. 1 and 2). The patients were followed up for a mean period of 21.5 (range 12 to 29) months. All cases underwent conventional radiography, MRI, CT, and bone scintigraphy. At presentation, the patients had a mean MSTS (Musculoskeletal Tumor Society) score of 60.8% (range 26 to 86.6%) and VAS (visual analog scale) score of 8 (range 2 to 10). Based on the decision of the tumor council, tissue biopsy and culture was performed on 12 patients due to the probability of a tumor. The patients were followed up on monthly basis with MR scans at presentation and later visits.

**Table 1:** Study data

Patient no.	Age (years)	Sex	Side	Biopsy	Biopsy outcome	Time to healing	Healing at the final MRI
1	7	F	Right	+	Partially necrotic, degenerative bone tissue	2 months	+
2	34	M	Left	+	Partially necrotic, degenerative bone tissue	3 months	+
3	38	F	Right	+	Osteonecrosis	4 months	+
4	13	M	Right	+	Osteomyelitis	3 months	+
5	8	F	Left	+	Osteonecrosis	2 months	+
6	16	M	Left	+	Osteonecrosis	3 months	+
7	30	F	Left	+	Osteonecrosis	3 months	+
8	30	F	Left	+	Sclerotic, osseous tissue	3 months	+
9	51	F	Left	+	Sclerotic, osseous tissue	4 months	+
10	24	F	Left	+	Partially necrotic, degenerative bone tissue	3 months	+
11	28	M	Left	+	Osteomyelitis	3 months	+
12	6	F	Left	+	Osteonecrosis	3 months	+
13	34	M	Bilateral	−	–	3 months	+
14	8	M	Right	−	–	4 months	+
15	35	F	Left	−	–	6 months	+
16	7	M	Bilateral	−	–	3 months	+

## Results

No abnormal findings suggesting osteomyelitis were detected in the laboratory tests or infection parameters of the patients. The CT scans showed no signs of a fracture line. Biopsies of the 12 patients revealed findings of necrotic bone tissue in three, osteonecrosis in five, sclerotic bone in two, and findings similar to those of osteomyelitis in two patients. No growth was observed in tissue cultures from the biopsy samples. Pain complaints of the 12 biopsied patients decreased significantly in the postoperative early term after a mean period of 10 days. Similarly, their MRI findings regressed after a mean period of 3 (range 2 to 4) months. The complaints and MRI findings of the four patients who did not have a biopsy regressed after a mean period of four (range 3 to 6) months. The mean MSTS score of the patients was 73.3% (range: 53.3% to 100%), and the mean VAS score was 5 (range 0 to 8) at the 6th month follow-up.

## Discussion

It is critical to consider the similarity in the clinical and radiological findings of a tumor or infection, make a differential diagnosis, and exhibit the challenges in the treatment of painful diaphyseal edemas of the tibia. Transient bone marrow edema is a self-limiting pathology defined with several terms, such as algodystrophy or transient osteoporosis syndrome, and is mostly seen in the hips, knees, ankles, and feet [6]. In our study, a case series with transient bone marrow edema confined to the diaphyseal region of the tibia, with limited reports in the literature and in which probabilities of tumor or infection, were excluded based on laboratory, clinical, and pathological findings were evaluated.

The etiology of the bone marrow edema remains unclear. While some authors suggest that the mechanisms of a stress fracture play a role in the condition and the altered biomechanics is the etiological factor [7, 8], the majority asserts that the etiological factor is ischemic. These authors think that the ischemia, due to asymptomatic intravenous thrombosis, causes necrosis in adipocytes, and that a decrease in the number of hematopoietic cells, and the reactive hyperemia and vasodilatation seen with the restoration of the blood supply lead to bone marrow edema syndrome and pain [9].

Bone marrow edema syndrome has recently begun to be considered a part of the pathophysiology of many different diseases. Accurate differential diagnosis of the primary pathology is necessary for proper patient management [10]. Twelve of our patients underwent biopsy for differential diagnosis.

Pain in patients with bone marrow edema develops following the irritation, or damage to the sensory nerves in the neurovascular structures of the bone marrow and the damage to the nervous tissue is a result of a tumor, trauma, or other external cause [11]. All patients presented to our clinic with complaints of localized non-traumatic pain on the tibia.

Laboratory tests’ results of bone marrow edema syndrome patients are normal. Direct radiographs exhibit no symptoms in the early term; however, local osteopenia may be seen in the late term. The diagnosis is made using MRI. Particularly in fat-suppressed sequences, T1-weighted sequences demonstrate low signal intensity and T2-weighted sequences show high signal intensity. Our patient series had all these MRI findings mentioned above and their laboratory results conformed with those in the literature.

The classical definition of bone marrow edema in the literature is a painful disease usually observed in a single bone in patients 30 to 60 years of age. The condition may be observed in the last trimester of pregnancy in patients 20 to 40 years old [12, 13]. There were nine females among our patients, but none of them was pregnant. The mean age of these patients was 23, a finding much lower than that reported in the literature.

The male:female ratio in the bone marrow edema syndrome is 3:1. While a rapid increase in pain and limitation in movements are seen in the first month, the severity of the symptoms remains the same in the following 2 months. After the third month, the symptoms start to recede and MR images show improvement [14, 15]. The symptoms of the 12 biopsied patients started to diminish dramatically in the postoperative early term. The MR findings have receded after a mean of 3 months. On the other hand, the symptoms in our patients who had not undergone biopsy continued for the first 2 months, as reported in the literature, and the MR images showed improvements after a mean of 4 months.

While intracellular and extracellular fluid retention, fat cell destruction, new bone formation, and fibrovascular regeneration are observed upon histological examination of the bone marrow edema [16, 17], the presence of osteoporosis or osteonecrosis were not reported [18, 19]. In our series, three patients had partially necrotic tissue, five had osteonecrosis, and two had sclerotic bone. The findings of two other patients were similar to those of osteomyelitis. Tissue cultures of these two patients showed no growth of pathogen. The condition was thus thought to be nonbacterial osteomyelitis. Chronic nonbacterial osteomyelitis (CNO) is an autoinflammatory bone disorder. Its clinical course may vary, and a single asymptomatic bone lesion may be observed. The diagnosis is often missed due to this varying course with mild and non-specific symptoms. The pathophysiology of the disease is still not fully comprehended [20]. Usually, a biopsy of the lesion is not necessary; however, it may be required in non-specific cases for distinguishing it from bone neoplasm. Non-steroidal anti-inflammatory drugs (NSAIDs) are used for first-line treatment [21].

Conservative methods and core decompression are recommended in the treatment of bone marrow edema syndrome. Usually, NSAIDs are used to alleviate pain and weight-bearing is restricted. The pain has been shown to lessen following decompression [22] and resolve after surgery. In a comparison between conservative treatment and decompression patients, Radke et al. found that the clinical outcomes were similar in both groups; however, patients who had undergone surgery showed faster recovery [23]. Our biopsied patients showed faster recovery in the early term than those who had not undergone biopsy, due to its core decompression effect.

Recently, vasoactive prostacyclin derivatives have been used in bone marrow edema patients to dilate the arterioles and venules. In a study comparing the effects of vasoactive prostacyclin derivatives and decompression, both groups showed a fast clinical recovery, whereas the patients treated with vasoactive prostacyclin derivatives showed a faster healing of the bone marrow edema [24]. The follow-up course of our patients who had not undergone a biopsy included administration of NSAIDs and restriction of weight-bearing.

Its retrospective design and the limited number of patients are the limitations of our study. However, our evaluation of the bone marrow edema in a reportedly rare location of tibial diaphysis, the challenges in diagnosis, and the treatment results with an adequate follow-up period renders our study remarkable.

## Conclusion

In conclusion, transient medullary bone edema of the tibial diaphysis with non-traumatic pain is reported in the literature over a limited number of cases. We concluded that biopsy is essential in this patient group as its decompression effect enabled a faster recession of complaints and radiological findings in the early term, in comparison with those who had not undergone a biopsy.
